# Mechanical and Electrical Properties of Polyethylene Terephthalate Glycol/Antimony Tin Oxide Nanocomposites in Material Extrusion 3D Printing

**DOI:** 10.3390/nano14090761

**Published:** 2024-04-26

**Authors:** Markos Petousis, Nikolaos Michailidis, Vassilis Saltas, Vassilis Papadakis, Mariza Spiridaki, Nikolaos Mountakis, Apostolos Argyros, John Valsamos, Nektarios K. Nasikas, Nectarios Vidakis

**Affiliations:** 1 Department of Mechanical Engineering, Hellenic Mediterranean University, 71410 Heraklion, Greece; markospetousis@hmu.gr (M.P.); mspyridaki@hmu.gr (M.S.); mountakis@hmu.gr (N.M.); valsamos@hmu.gr (J.V.); 2 Physical Metallurgy Laboratory, Mechanical Engineering Department, School of Engineering, Aristotle University of Thessaloniki, 54124 Thessaloniki, Greece; nmichail@auth.gr (N.M.); aargyros@auth.gr (A.A.); 3 Centre for Research & Development of Advanced Materials (CERDAM), Centre for Interdisciplinary Research and Innovation, Balkan Centre, Building B’, 10th km Thessaloniki-Thermi Road, 57001 Thessaloniki, Greece; 4 Department of Electronic Engineering, Hellenic Mediterranean University, 73133 Chania, Greece; saltas@hmu.gr; 5 Institute of Electronic Structure and Laser of the Foundation for Research and Technology-Hellas (IESL-FORTH)–Hellas, N. Plastira 100m, 70013 Heraklion, Greece; v.papadakis@uniwa.gr; 6 Department of Industrial Design and Production Engineering, University of West Attica, 12243 Athens, Greece; 7 Division of Mathematics and Engineering Sciences, Department of Military Sciences, Hellenic Army Academy, Vari, 16673 Attica, Greece; nasikas@sse.gr

**Keywords:** polyethylene terephthalate glycol (PETG), Antimony Tin Oxide (ATO), material extrusion (MEX), 3D printing, nanocomposites, materials characterization

## Abstract

In this study, poly (ethylene terephthalate) (PETG) was combined with Antimony-doped Tin Oxide (ATO) to create five different composites (2.0–10.0 wt.% ATO). The PETG/ATO filaments were extruded and supplied to a material extrusion (MEX) 3D printer to fabricate the specimens following international standards. Various tests were conducted on thermal, rheological, mechanical, and morphological properties. The mechanical performance of the prepared nanocomposites was evaluated using flexural, tensile, microhardness, and Charpy impact tests. The dielectric and electrical properties of the prepared composites were evaluated over a broad frequency range. The dimensional accuracy and porosity of the 3D printed structure were assessed using micro-computed tomography. Other investigations include scanning electron microscopy and energy-dispersive X-ray spectroscopy, which were performed to investigate the structures and morphologies of the samples. The PETG/6.0 wt.% ATO composite presented the highest mechanical performance (21% increase over the pure polymer in tensile strength). The results show the potential of such nanocomposites when enhanced mechanical performance is required in MEX 3D printing applications, in which PETG is the most commonly used polymer.

## 1. Introduction

Additive manufacturing (AM), widely termed as 3D printing, is a novel and rapidly expanding manufacturing technology that is suitable for a variety of applications that require complex geometries for manufactured products and has transformed manufacturing processes [1]. Three-dimensional printing technology is an AM technique that provides the fabrication of various shaped items in a short period of time while maintaining a low cost [2] when compared with conventional manufacturing techniques and processes. The usefulness of 3D printed parts for engineering purposes depends strongly on the detailed knowledge of their mechanical properties. All 3D printed parts made using the material extrusion (MEX) method were fabricated by feeding a filament into a 3D printer nozzle, out of which the material was extruded. During extrusion, the material flows in a viscous condition after being heated at temperatures close to and above its melting temperature, T_m_, and is then deposited into a layer-by-layer structure while it is immediately hardened [3,4] as it cools off at temperatures below T_m_. Via the process described above, various three-dimensional (3D) parts derived from models designed using computer-aided design (CAD) data [5] are manufactured and can be utilized in several applications in major industries such as defense, aerospace [6,7,8,9,10,11], aircraft [12], engineering [13], energy [14], electronics [15], medicine [16,17,18,19,20,21], food [22], and automobiles [7,8,23,24]. Seven groups of different AM methods have been described by the American Society for Testing and Materials (ASTM), including material extrusion [25], which was employed in this study. As mentioned previously, significant efforts have been made to improve the mechanical characteristics of various fabricated 3D-P parts [26,27]. Therefore, it is important to optimize the 3D printing parameters [28] to achieve the latter. The most vital printing parameters are printing speed, temperature, layer thickness, infill density, and air gaps [29,30]. Additionally, according to the literature, nozzle temperature can influence the fluidity and solidification characteristics of extruded filaments [31].

Poly(ethylene terephthalate) (PETG) is an amorphous linear polymeric material suitable for extrusion, thermoforming, injection, and blow molding [32,33]. PETG is highly transparent [34] because of its low crystalline-phase content. It has adequate mechanical properties, chemical resistance [35,36], high ductility [37], and heat resistance, and is an inexpensive and versatile biomaterial [38]. It is derived from Polyethylene Terephthalete (PET) polymer, filled with glycol [39], and amongst other characteristics, it exhibits good chemical alkali resistance, high shrinkage, transparency, gloss, low haze, and good printability [40]. There are various applications in the medical field, such as tissue engineering, dentistry, optometry, vascular health, cardiology, orthopedics, neurology, gynecology, and surgery [38] in which it is used, as its properties fit many of the requirements for various applications in the fields mentioned above. The mechanical performance of MEX 3D printing has been reported for different types of tests [2,41,42,43,44]. It has also been used as a matrix for the development of composites with carbon-based fillers [45,46,47,48]. Much research has been conducted on the sustainability of polymers owing to their very good response when recycled [40,49,50].

Ceramics are known for their exceptional properties, such as high strength and resistance to wear [51]. Owing to these properties, they are common materials in demanding applications such as coatings [52,53], cutting tools [54,55], armor, and ballistic applications [56,57]. They are often used as fillers in polymeric matrices to improve performance [58,59,60,61]. Lately, their use and the research on their performance as fillers in MEX 3D printing. Ceramics, such as silicon nitride [62,63], boron carbide [64], titanium carbide [65], titanium nitride [66], and tungsten carbide [67], have been tested on different polymeric matrices. Antimony Tin Oxide (ATO) is a ceramic filler that is widely used in electrical applications owing to its electrical conductivity [68,69,70]. Therefore, it has mostly been investigated as a filler with the aim of examining the dielectric properties and control of its conductivity when incorporated into various polymeric matrices [71,72,73]. It is also characterized by a variety of other properties, including low resistivity, good optical transmission, high surface composition, and catalytic activity, which vary according to the applied technique of nanoparticle preparation [74,75,76] when ATO is prepared as nanoparticles. ATO is also considered a transparent conductive oxide characterized by high transparency in the visible range and considerable electrical conductivity [77,78,79,80,81]. ATO is useful in applications such as flat-panel displays, solar cells, and electromagnetic interference shielding [78,82,83,84]. Their optical properties make them suitable for use in films [85,86].

In this work, various composites were created by combining PETG as the matrix material with ATO as the filler in an effort to examine their performance via a variety of tests. The effect of ATO nanoparticles as reinforcement agents for the PETG thermoplastic was evaluated. The aim was to improve the mechanical performance of the PETG thermoplastic in MEX 3D printing, following a process that can be easily industrialized. Thus, the robustness of parts in applications in which PETG thermoplastic is the suitable polymer to use will be improved, and the potential for expanding its use can be enabled. Popular applications that 3D printed PETG composites can be applied to are in the aeronautical, automotive, and food safety sectors [87]. PETG/ATO nanocomposites, to the authors’ best knowledge, have not been investigated in the literature so far for their mechanical properties, let alone in the AM or MEX 3D printing technologies, and by considering the ease of industrialization of the process, as mentioned above.

Mixtures of five different filler percentages were produced: PETG/ATO 2.0, 4.0, 6.0, 8.0, and 10.0 wt.%, respectively. First, the materials were thoroughly mixed before being converted into filaments. The extruded filaments were then supplied to a 3D printer to manufacture specimens. The same procedure was followed for the production of the corresponding pure PETG samples for utilization as reference points to compare the results derived from the conducted tests. The mechanical properties were examined using tensile and flexural microhardness and toughness tests, respectively. Their thermal characteristics were determined using thermogravimetric analysis (TGA) and differential scanning calorimetry (DSC). Raman analysis was conducted to determine their structural characteristics. The electrical/dielectric properties were investigated over a broad frequency range, as it was expected that ATO conductive nanoparticles would modify the electrical properties of the nanocomposites, according to existing reports in the literature. Scanning electron microscopy (SEM) and energy-dispersive spectroscopy (EDS) were used to examine the morphological and structural characteristics of the samples. Micro-computed tomography (μ-CT) was employed to examine the dimensional accuracy and internal structure (to locate defects, voids, etc.) of the samples. The developed nanocomposites showed that ATO has potential because its mechanical properties were improved by the nanocomposite, with 6 wt.% loading being the most efficient. However, the electrical conductivity did not improve significantly. The main objectives of this study were as follows:

- Reveal the efficiency of ATO as a reinforcement agent in the PETG polymer.
- Investigate the ability of ATO to modify the electrical properties of the PETG polymer.
- Propose PETG/ATO nanocomposites for the MEX 3D printing process with enhanced performance. To improve the efficiency of applications, the PETG polymer is used overall, further expanding its use.

## 2. Materials and Methods

**Figure 1:**
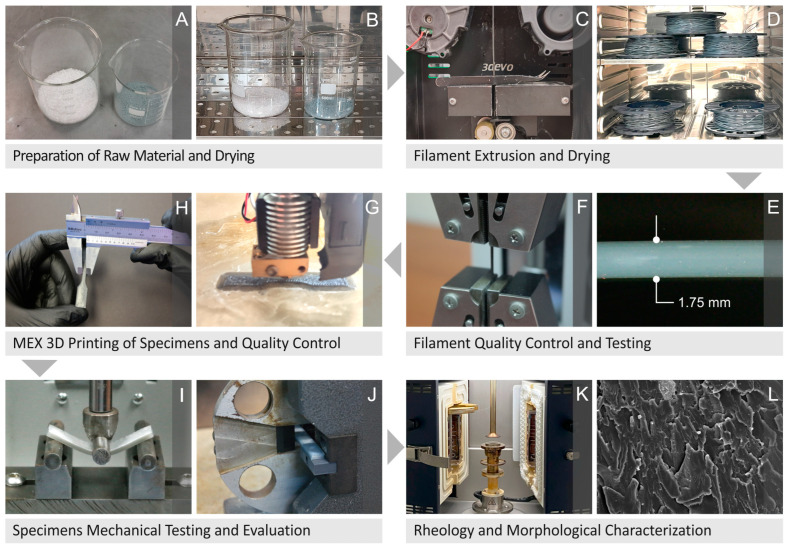
(**A**,**B**) Preparation procedure and drying of the raw materials, (**C**–**F**) filament extrusion, drying, inspection of quality and mechanical properties test, (**G**,**H**) manufacturing of the 3D-P specimens via MEX and inspection of their quality, (**I**,**J**) mechanical testing and evaluation of the specimens, as well as (**K**,**L**) their rheology and morphological characterization.

To conduct this investigation, a series of initial assessments were performed on the raw materials, filaments, specimens, and tests of the samples. Figure 1A,B show the preparation procedure of the raw materials in proper quantities for the tests, followed by drying in an oven. Figure 1C–F depict the extrusion of the filaments and their drying, respectively, before quality control and testing of their mechanical properties. Figure 1G,H show the MEX 3D-P and quality inspection of the specimens, respectively, while Figure 1I–L show the mechanical testing, evaluation, rheological, and morphological characterization of the specimens, respectively.

### 2.1. Materials

The employed Polyethylene Terephthalate Glycol (PETG) was purchased from Felfil Srl (Torino, Italy) in pellets. The ATO spherical nanoparticles (NPs) (7–11% Sb_2_O_5_ to SnO_2_) were supplied by Sigma-Aldrich (St. Louis, MO, USA). According to the manufacturer, the particle size is <50 nm and the surface area is 47 m^2^/g.

### 2.2. Preparation of the PETG and PETG/ATO Filaments and 3D Printing of the Specimens

First, appropriate quantities of starting materials were prepared and mixed using a mechanical homogenizer (500 W, 30 min, 4000 rpm, no solvents or binding agents were used), which created five (5) different mixtures of 2.0, 4.0, 6.0, 8.0, and 10.0 wt.% filler quantities. The mixtures were then left in an oven overnight (8 h) at 80 °C to dry before executing the filament extrusion procedure using a single-screw 3D Evo Composer 450 extruder (3devo B.V., NL). This extruder features a filament–diameter sensor that operates during filament production. It then proceeds to micro-adjust the extrusion speed, if needed, to maintain a steady diameter filament. The 3devo Composer Extruder also comes with a specifically engineered screw design crafted to efficiently blend materials and nano additives, as per the manufacturer’s specifications.

The range of filler percentages was determined by gradually increasing the filler content and testing the corresponding samples. When the mechanical properties started to decline, the filler loading did not further increase, indicating the saturation of the filler in the matrix. The optimum extrusion parameters were set according to information provided in the literature [40]. All the fabricated filaments measured a diameter between 1.65 mm and 1.85 mm, which was adequate for 3D printing. Prior to the 3D printing procedure, the filament underwent quality control and was oven-dried overnight at 80 °C. An FFF 3D printer, Intamsys Funmat HT 3D from Intamsys Technology Co., Ltd. (Shanghai, China), was used for the fabrication of pure PETG and PETG/ATO specimens. The design for all 3D-P specimens intended for mechanical testing was created using 3D Autodesk^®^ Fusion 360™ (Autodesk^®^, Inc., San Francisco, CA, USA) software before exporting the design into files of standard tessellation language (STL) form.

### 2.3. SEM and EDS Analysis of ATO and the Nanocomposites’ Specimens

An electron microscope (Jeol JSM-IT700HR field-emission SEM, Jeol, Tokyo, Japan) was used to obtain scanning electron microscopy (SEM) images. Images were captured from both the sides and fracture surfaces of the pure PETG and PETG/ATO nanocomposite specimens. EDS was used to investigate the chemical composition of the samples and was conducted using the same device as that used for SEM. The ATO material was subjected to these tests to validate its specifications. To conduct the above analyses, a high-vacuum mode and an acceleration voltage of 5 kV were used while the samples were gold-sputtered. SEM and EDS mapping images from the ATO nanoparticles are available in the Supplementary Materials of the study.

### 2.4. Micro-Computed Tomography (μ-CT)

For the investigation of the porosity as well as the dimensional deviation of the fabricated samples, microcomputed tomography (μ-CT) scanning was employed and conducted by a Tomoscope HV Compact 225  kV Micro Focus CT-scanner (Werth Messtechnik GmbH, Giessen, Germany) of a 1024 × 1024-pixel sensor. VG Studio MAX 2.2 software (Volume Graphics GmbH, Heidelberg, Germany) was used to process the derived data. These investigations were aimed at evaluating the effects of the additives on the 3D-P structure. The dimensional accuracy was examined with a 75 L setup (72.58 μm resolution on the *X*-axis and 72.65 μm resolution on the *Y*-axis). The porosity was examined with a 16 L setup (15.46 μm resolution on the *X*-axis and 15.49 μm resolution on the *Y*-axis). In both cases, 1600 sections were acquired per revolution.

### 2.5. Mechanical Characterization

Tensile, flexural, Charpy impact, and microhardness (M-H) tests were performed to investigate the mechanical performance of the specimens. ASTM D638-02a for V-type tensile specimens (3.2 mm thick) was followed for the manufacturing and tensile tests. A tension/flexure test apparatus in tensile mode, Imada MX2 (Imada Inc., Northbrook, IL, USA), with standardized grips, was used for tensile testing. For the flexural 3-point bending testing, ASTM D790-10 (3-point bending test with a 52.0 mm support span) was considered, while the machine utilized was the same as for the tensile test, but in the flexural mode setup, in accordance with the corresponding standard. Impact testing was based on ASTM D6110-04 and conducted using a Terco MT 220 (Terco, Kungens Kurva, Sweden) Charpy impact device. For microhardness testing, the measurements were taken in accordance with ASTM E384-17, using an Innova Test 300 Vickers device from Innovatest Europe BV, Maastricht, The Netherlands, on a fully polished surface of the specimens. A force of 100 gF was applied, and an indentation duration of 10 s was set.

**Figure 2:**
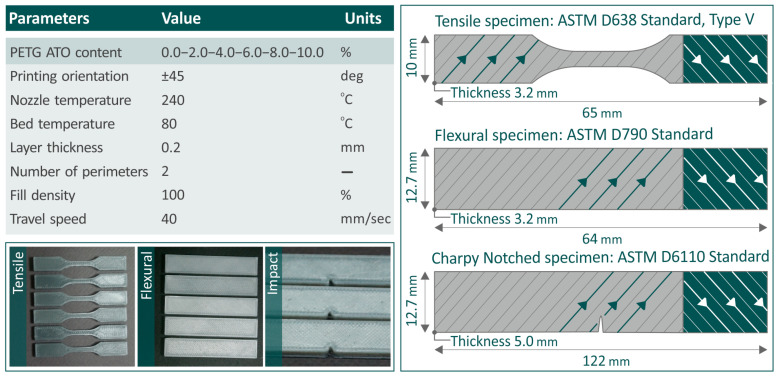
The 3D-P parameters of the specimens, samples from the tensile flexural and impact specimens, and their models, along with dimensions according to which their fabrication was conducted.

Figure 2 presents the values of the parameters set during the 3D-P of the specimens. The nozzle temperature was 240 °C, the bed temperature was 80 °C, the layer thickness was 0.2 mm, and the print speed was 40 mm/s. Figure 2 shows some samples from the 3D-P tensile, flexural, and impact specimens, as well as the dimensions based on which the 3D specimens were fabricated.

### 2.6. Raman Spectroscopy Analysis and Fourier Transform Infrared (FTIR) Spectroscopy

The produced samples were analyzed via Raman spectroscopy using a HORIBA Scientific confocal LabRAM HR Raman Spectrometer (Kyoto, Japan). The main 532 nm laser line of the spectrometer was used at a power of 90 mW. To reduce laser power on the sample in an effort to eliminate possible alterations caused on the sample by an intense focused laser beam, a 5% Neutral Density filter was used inside the optical path. The objective lens used for sample excitation and imaging was a 50× Olympus microscope objective lens (LMPlanFL N) with a numerical aperture (NA) of 0.5- and 10.6 mm working distance. The microscope settings for the data acquisition were as follows:

- Acquisition spectral range from 50 up to 3900 cm^−1^;
- Spectrometer grating 600 grooves/mm, resulting in 2 cm^−1^ spectral resolution;
- Sample exposure time is 10 s at each measurement point;
- We acquired a total of five accumulations at each point for statistical purposes and to improve the signal-to-noise ratio, which in turn would yield high-quality Raman spectra;
- Imaging resolution was 1.7 μm lateral and 2 μm axial.

At the sample’s surface, the measured laser power was 2 mW.

Following the data acquisition, the measured areas were visually inspected using a microscope to ensure the absence of discoloration or degradation due to laser irradiation.

Raman’s raw data were then processed using the LabSpec v. 6 software (HORIBA, Kyoto, Japan). Each spectrum acquired was processed with the same methodology:

(a) Cosmic rays were removed; (b) signal denoise with 5-point kernels; (c) background removal using a 6th-grade polynomial; (d) spectra were recalibrated by the maximum peak; (e) data normalization by a unit vector; (f) from each spectrum, the PETG Pure spectrum was subtracted.

The ATR/FT-IR measurements, which determined absorbance, were conducted using a Bruker Vertex 70v FT-IR (Bruker, Billerica, MA, USA) vacuum spectrometer. This spectrometer was equipped with a single reflection diamond crystal A225/Q Platinum ATR unit, and the samples were analyzed via total reflection measurements in the spectral range of 7500–350 cm^−1^. Interferograms were recorded at a resolution of 4 cm^−1^ (8 scans) and were apodized with a Blackman–Harris function. Fourier transformation was then performed with two levels of zero filling to obtain spectra encoded at 2 cm^−1^ intervals. The measurements were noise-corrected via automatic subtraction of a diamond crystal background signal. To ensure accuracy, the samples were carefully placed under the ATR press, and after each measurement, the sample area and the tip of the A225/Q ATR unit were cleaned with pure ethanol (Et-OH; Sigma-Aldrich, Munich, Germany) and left to dry for several minutes.

### 2.7. Rheometric Performance Examination

A DHR-20 Discovery Hybrid Rotational Rheometer (TA Instruments, New Castle, DE, USA) was used for rheometric measurements according to ASTM D1238-13 for the melt flow rate (MFR). The device consists of an Environmental Test Chamber with a parallel-plate setup for temperature regulation. The acquisition time was 10 s at each measurement point to prevent excessive heating and decomposition. The flow rates of the materials at certain temperatures and previously determined pressures were assessed using the melt flow rate (MFR) and rotational rheometric tests.

### 2.8. Thermal Properties Examination

Thermogravimetric analysis (TGA) was performed along with differential scanning calorimetry (DSC) to obtain information on the thermal properties of the pure and nanocomposite samples. A Diamond Perkin Elmer device (Waltham, MA, USA) was employed for TGA analysis (40–550 °C temperature cycle, 10 °C/min temperature increase rate). DSC analysis was conducted using a Discovery Series DSC-25 DSC calorimeter (TA Instruments, New Castle, DE, USA). The device is equipped with an RSC-90 Refrigerated Cooling System (TA Instruments, New Castle, DE, USA). During both the TGA and DSC experiments, an inert environment and high-purity N_2_ (nitrogen gas) were present.

### 2.9. Broadband Dielectric Spectroscopy (BDS) Measurements

BDS measurements of the PETG/ATO composites in the frequency range of 10^−2^ Hz to 4 MHz were conducted using an Alpha-ANB high-resolution dielectric analyzer in conjunction with a ZGS Alpha Active sample holder and a BDS 1100 module (Novocontrol Technologies GmbH & Co., Montabaur, Germany). The two-electrode configuration of the sandwich-structured capacitor was implemented on disk-shaped specimens (diameter: 40 mm, thickness: 4 mm) prepared by thermal pressing. The conductive paste was applied to both sides of the specimen to guarantee effective electrical contact with the gold-plated electrodes of the sample holder. An AC voltage with a root-mean-square (Vrms) value of 1 V was applied, and the measurements were conducted at ambient temperature. Control and data acquisition were performed using WinDeta v. 5.8 software (Novocontrol Technologies GmbH & Co., Montabaur, Germany). The dielectric properties are represented as complex functions of the dielectric permittivity (ε*(ω)), dissipation factor (tan(δ)), and AC conductivity (σ*(ω)), as expressed by the following equations:

(1) $$\varepsilon^{*}\left(\omega \right)=\varepsilon^{\prime }-i\varepsilon^{\prime \prime }=\frac{C\left(\omega \right)}{\varepsilon_{ο}\pi r^{2}/d}-i\frac{1}{\omega R\left(\omega \right)\varepsilon_{ο}\pi r^{2}/d}$$

(2) $$\tan\left(\delta \right)=\frac{\varepsilon^{\prime \prime }}{\varepsilon^{\prime }}$$

and

(3) $$\sigma^{*}\left(\omega \right)=\sigma^{\prime }-i\sigma^{\prime \prime }=i\omega \varepsilon_{ο}(\varepsilon^{*}\left(\omega \right)-1)=\omega \varepsilon_{ο}\varepsilon^{\prime \prime }-i\omega \varepsilon_{ο}\left(\varepsilon^{\prime }-1\right)$$

where the resistance R(ω) and the capacitance C(ω) are the measured quantities of the dielectric analyzer, d is the specimen thickness, r is its radius, ω=2πf is the angular frequency, and $\varepsilon_{ο}$ is the permittivity of the vacuum. The above functions are widely used in the dielectric characterization of materials because the real part of the complex permittivity (ε′) measures the energy storage in the material under the effect of an electric field, whereas the dissipation factor (tan(δ)) $\varepsilon^{\prime \prime }$ is related to the energy loss within the material.

## 3. Results

### 3.1. Raman Spectroscopy Characterization

**Figure 3:**
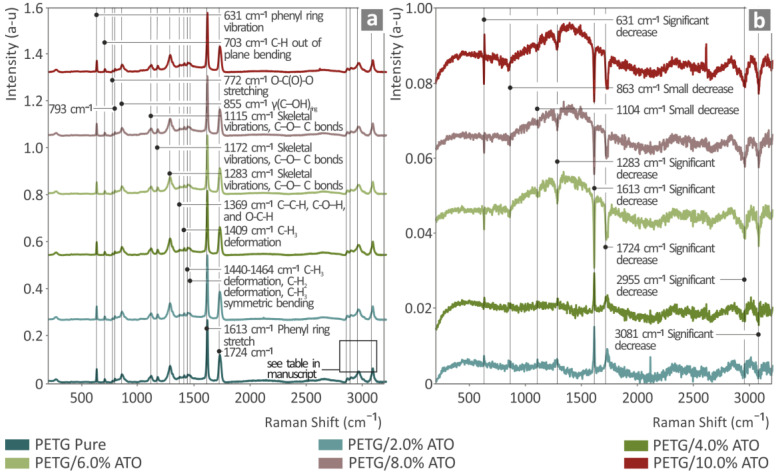
(**a**) Raman spectra of pure PETG, PETG/2.0 wt.% ATO, PETG/4.0 wt.% ATO, PETG/6.0 wt.% ATO, PETG/8.0 wt.% ATO, and PETG/10.0 wt.% ATO; (**b**) results after subtracting pure PETG from all the PETG/ATO composites.

Figure 3a shows the Raman spectra of the pure PETG/ATO composites (2.0, 4.0, 6.0, 8.0, and 10.0 wt.%), while Figure 3b shows the curves from subtracting pure PETG from all the composites. The Raman peaks corresponding to the pure PETG sample were obtained from the literature and are listed in Table 1. As shown, as the concentration of ATO increased in the PETG, the related Raman lines of pure PETG lost their signal intensity. Furthermore, the broad photoluminescence appeared to increase as the ATO concentration increased from 800 to 1800 cm^−1^. The addition of ATO to PETG resulted in a decrease in some Raman lines, probably owing to the photoluminescence of ATO. The Raman lines that decreased in intensity are listed in Table 2.

**Figure 4:**
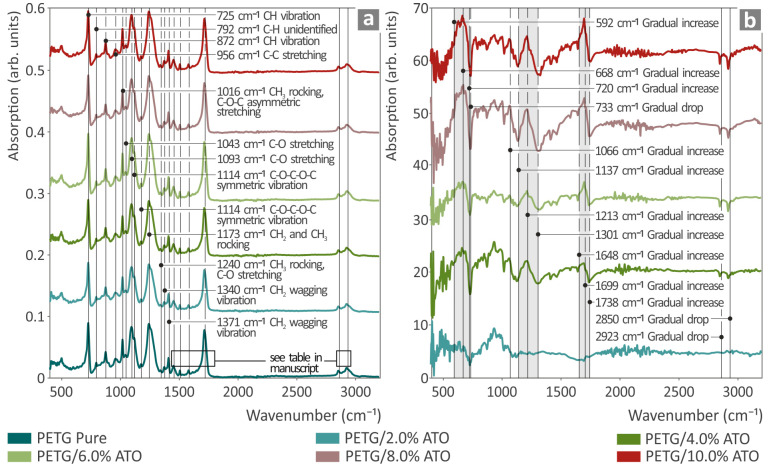
(**a**) ATR/FTIR spectra from pure PETG, PETG/ATO 2 wt.%, PETG/ATO 4 wt.%, PETG/ATO 6 wt.%, PETG/ATO 8 wt.%, and PETG/ATO 10 wt.%, (**b**) ATR/FTIR spectral differences in PETG/ATO 2 wt.%, PETG/ATO 4 wt.%, PETG/ATO 6 wt.%, PETG/ATO 8 wt.%, and PETG/ATO 10 wt.% from PETG/pure.

In Figure 4, we can observe the clear ATR/FTIR spectra from the pure material PETG. The related ATR/FTIR peaks were identified and are shown in the next Table 3 together with their validation by the literature. The addition of ATO in PETG presented three ranges with a gradual increase in absorption. Particularly, the ranges were 592–720 cm^−1^ with a peak at 668 cm^−1^; 1137–1301 cm^−1^ with a peak at 1213 cm^−1^; and 1648–1738 cm^−1^ with a peak at 1699 cm^−1^. To the contrary, the bands at 733, 2850, and 2923 cm^−1^ presented a gradual drop. All those differences are shown in the next Table 4.

### 3.2. Thermal Properties Characterization via TGA and DSC

**Figure 5:**
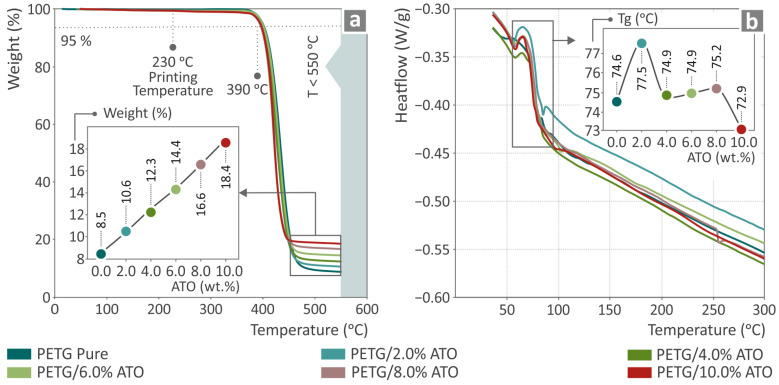
Results from the thermal properties investigation of neat PETG and PETG/ATO (2.0, 4.0, 6.0, 8.0, and 10.0 wt.%) composites by (**a**) TGA and (**b**) DSC graphs.

The results of the investigation of the thermal properties of the samples are shown in Figure 5a,b using the TGA and DSC curves of pure PETG and PETG/ATO 2.0, 4.0, 6.0, 8.0, and 10.0 wt.%. The performance of the composite samples did not exhibit any major differences. The inset graph in Figure 5a indicates the residual weight with respect to the filler percentage of the composites, showing that the higher the filler percentage, the higher the residual weight. The TGA graphs also verified that the extrusion temperatures used throughout the study did not cause any degradation effects on the tested samples, which might have affected their performance. Figure 5b shows the heat flow as a temperature DSC graph of neat PETG and all PETG/ATO composites, along with an inset graph showing the glass transition temperatures (T_g_) of all the composites. It can be observed that only the PETG/2.0 wt.% ATO, the glass transition temperature presents an importantly increased value in relation to pure PETG, while the T_g_ value of PETG/10.0 wt.% ATO is the only one much lower than that of pure PETG.

### 3.3. Viscosity and Melt Flow Rate Results

**Figure 6:**
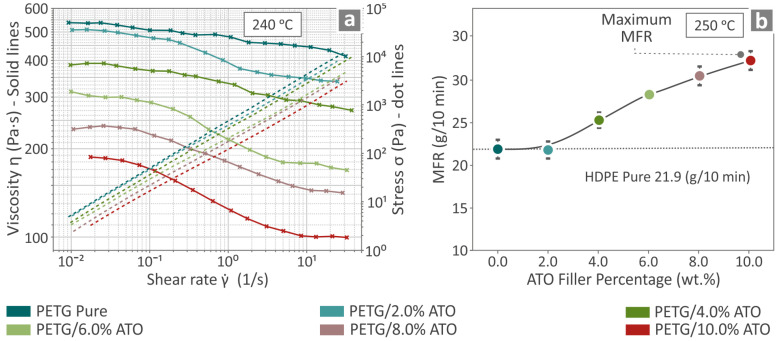
Results regarding neat PETG and the PETG/ATO (2.0, 4.0, 6.0, 8.0, and 10.0 wt.%) composites when tested for (**a**) viscosity and stress, as well as (**b**) MFR.

Viscosity and stress versus shear rate graphs for pure PETG and PETG/ATO 2.0, 4.0, 6.0, 8.0, 10.0 wt.%, at 240 °C are presented in Figure 6a. Figure 6b shows the MFR results for pure PETG and all PETG/ATO composites at 250 °C. The viscosity results indicated that, as the stress increased, the viscosity decreased, showing that the material has a shear thinning behavior sourced from the phenomenon that as the shear rate increases, the polymer’s chains tend to disentangle and thus result in a lower resistance to move which in this case is translated in lower viscosity values. Also, as the filler percentage increased, the viscosity and stress decreased because of the slippage phenomena appearing due to the incorporation of the hard filler in the polymer matrix, resulting in a reduction in chain strength against applied shear stresses. Also, the MFR measurements increased with the filler percentage, and the maximum value was found at the PETG/10.0 wt.% ATO concentration.

### 3.4. Filament Inspection and Quality Control

**Figure 7:**
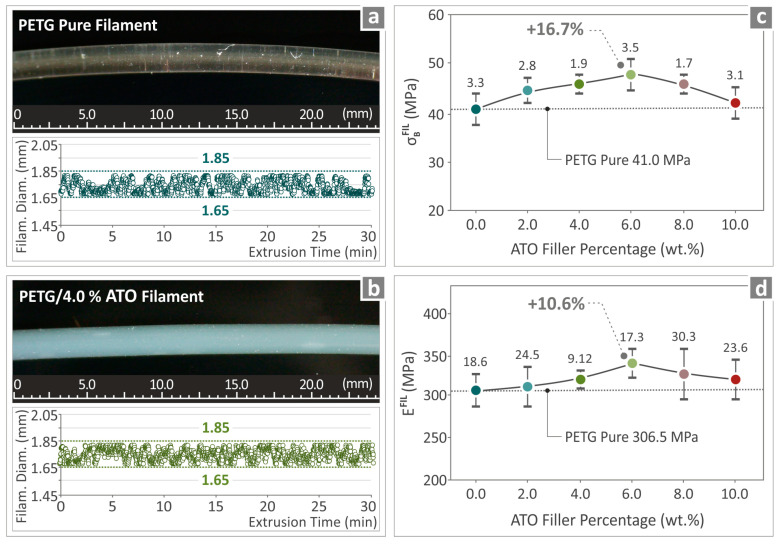
(**a**,**b**) Images of the fabricated pure PETG and PETG/4.0 wt.% ATO filaments and the monitoring of their diameters, (**c**) results from the tensile strength and (**d**) tensile modulus of elasticity of pure PETG, and all PETG/ATO composites.

Figure 7 shows the results of the quality inspection of the prepared filaments. Figure 7a,b show images of the fabricated pure PETG and PETG/4.0 wt.% ATO filaments, respectively, as well as the results from the recording of their diameter during their fabrication. Both filaments were characterized by smooth surfaces, while their diameters were kept steady between 1.65 mm and 1.85 mm. Figure 7c,d show, respectively, the values of the tensile strength and tensile modulus of elasticity of the pure PETG/ATO filament samples, respectively. It can be seen that the PETG/6.0 wt.% ATO presented the highest value of both tensile strength (16.7% above pure PETG) and tensile modulus of elasticity (10.6% above pure PETG).

### 3.5. Characterization of Specimens’ Mechanical Performance

**Figure 8:**
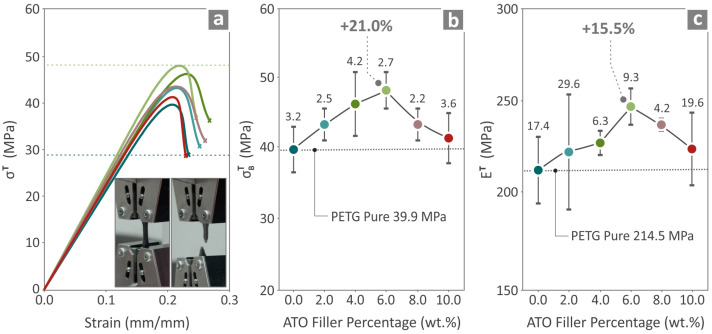
Neat PETG, PETG/2.0 wt.% ATO, PETG/4.0 wt.% ATO, PETG/6.0 wt.% ATO, PETG/8.0 wt.% ATO and PETG/10.0 wt.% ATO results from the tensile testing of the samples namely (**a**) tensile stress to strain graphs, (**b**) tensile strength, and (**c**) tensile modulus of elasticity.

The tensile properties of the pure PETG and PETG/ATO 2.0, 4.0, 6.0, 8.0, and 10.0 wt.% are presented in Figure 8. Figure 8a shows the tensile stress on the strain graph and the two images taken before and after testing a randomly chosen specimen. Figure 8b,c present the tensile strength and tensile modulus of elasticity results, respectively, where the highest values above those of pure PETG are found in PETG/6.0 wt.% ATO, 21.0%, and 15.5%, respectively.

**Figure 9:**
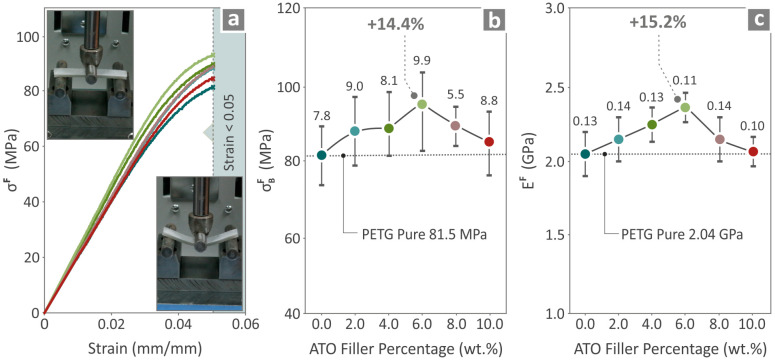
PETG pure, PETG/2.0 wt.% ATO, PETG/4.0 wt.% ATO, PETG/6.0 wt.% ATO, PETG/8.0 wt.% ATO and PETG/10.0 wt.% ATO results from the flexural properties testing of the samples namely (**a**) flexural stress to strain graphs, (**b**) flexural strength, and (**c**) flexural modulus of elasticity.

The flexural properties of pure PETG/*x*wt%ATO were *x* = 2.0, 4.0, 6.0, 8.0 and 10.0 wt.%, are shown in Figure 9. Figure 9a shows the flexural stress on the strain graph, accompanied by two images taken before and during the testing of a randomly chosen specimen. Figure 9b shows the flexural strength values for all composites; the highest value was observed in the case of PETG/6.0 wt.% ATO (14.4% increase with respect to pure PETG). The highest flexural modulus of elasticity was detected at PETG/6.0 wt.% ATO (15.2% increase with respect to pure PETG).

**Figure 10:**
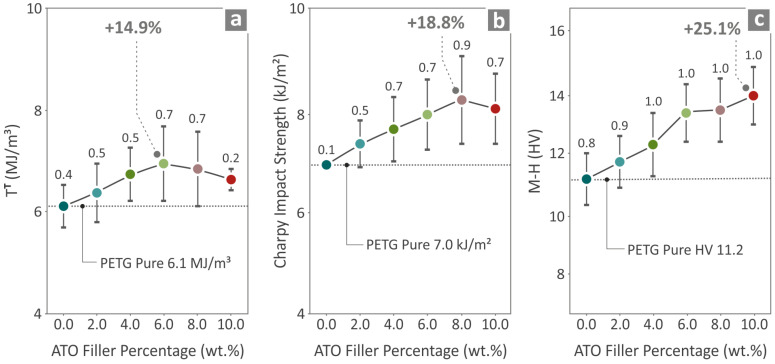
Results regarding the values of neat PETG and all the PETG/ATO composites as to the (**a**) tensile toughness, (**b**) Charpy impact strength, and (**c**) M-H.

Figure 10 shows the tensile toughness (Figure 10a), Charpy impact (Figure 10b), and microhardness (Figure 10c) of the pure PETG and PETG/ATO 2.0, 4.0, 6.0, 8.0, 10.0 wt.% composites. PETG/6.0 wt.% ATO presented the greatest value regarding tensile toughness, which was found to be 14.9% above pure PETG. The greatest Charpy impact was at PETG/8.0 wt.% ATO, 18.8% above pure PETG, while PETG/10.0 wt.% ATO presented the highest value of microhardness, 25.1% above PETG pure.

### 3.6. Electrical/Dielectric Characterization of PETG/ATO Composites’ Samples

**Figure 11:**
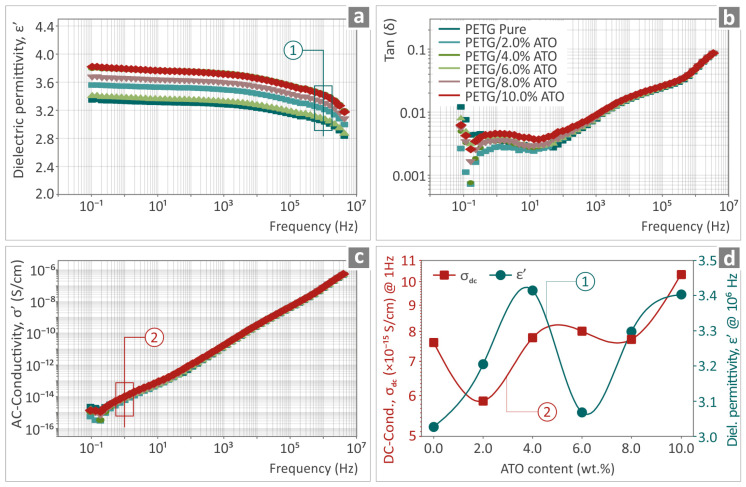
(**a**) The real part of dielectric permittivity (ε’), (**b**) the dissipation factor tan(δ), and (**c**) the real part of ac-conductivity (σ’) as a function of the frequency of pure PETG and PETG/ATO composites at various ATO filler contents (0–10% wt.). The variation in dc-conductivity, σ_dc_ (measured at 1 Hz), and dielectric permittivity, $\varepsilon_{\infty }$ (measured at 1 MHz) as a function of ATO content is shown in (**d**).

The real part of dielectric permittivity (ε’), the dissipation factor tan(δ), and the real part of ac-conductivity (σ’) in the measured frequency range of pure PETG and PETG/ATO nanocomposites at various filler contents (0–10% wt.) are shown in Figure 11a–c. The dielectric permittivity of pure PETG demonstrates minimal frequency dispersion across the measured frequency spectrum, i.e., ε’ increases gradually from 2.8 at 4 MHz to 3.3 at low frequencies, in agreement with previously reported values [103]. With increasing ATO content up to 10% wt., no significant spectral changes were observed over the entire frequency range, whereas $\varepsilon_{\infty }$ did not show any considerable change, varying from 2.8 to 3.4, as depicted in Figure 11d. This observation implies that the dielectric behavior of PETG/ATO composites is mainly determined by the properties of the polymer matrix.

The dissipation factor of pure PETG showed low dielectric loss values (<0.04) over a broad range of frequencies up to 4 kHz, while two distinct broad relaxation peaks centered around 2 Hz and 10 kHz were clearly observed. The high-*f* mechanism remains unaffected by the addition of conductive nanoparticles and is related to the relaxation polarization of the polymer matrix [104]. The low-*f* feature is affected by filler addition and should be attributed to the interfacial polarization caused by the interaction between the polymer matrix and conductive filler particles.

The conductivity spectra of pure PETG and PETG/ATO nanocomposites show the characteristic behavior of insulating materials such as polymers, that is, AC conductivity scales with *ω* at high frequencies. In the low-frequency range, a direct current (DC) plateau begins to develop, corresponding to dc-conductivity values of the composites varying by one order of magnitude around the value of 10^−15^ S/cm. The fluctuating behavior of the conductivity values measured at 1 Hz for various filler contents is shown in Figure 11d.

Based on the above findings, we may conclude that the influence of ATO nanoparticles on the overall electrical/dielectric behavior of PETG/ATO composites is negligible in the studied filler content, that is, up to 10 wt.%. The variation in either DC-conductivity or dielectric constant with increasing ATO content does not appear to show any particular trend and is rather determined by the random distribution and/or segregation of filler nanoparticles to the polymer matrix. The latter also contributes to weak changes in the interfacial polarization that takes place between the ATO nanoparticles and the matrix.

### 3.7. Micro-Computed Tomography of the Specimens

**Figure 12:**
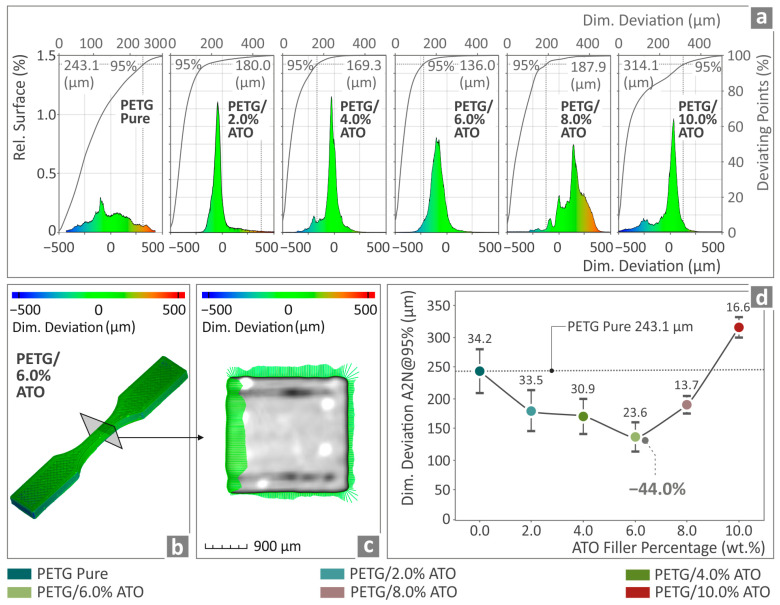
(**a**) Micro-computed tomography results regarding the dimensional deviation of pure PETG, PETG/2.0 wt.% ATO, PETG/4.0 wt.% ATO, PETG/6.0 wt.% ATO, PETG/8.0 wt.% ATO and PETG/10.0 wt.% ATO; (**b**,**c**) structural deviation of a PETG/6.0 wt.% ATO tensile specimen by color-coded mapping; (**d**) A2N dimensional deviation at 95% of pure PETG and all the PETG/ATO composites.

In Figure 12, the μ-CT scanning results for pure PETG and PETG/ATO 2.0, 4.0, 6.0, 8.0, 10.0 wt.% are presented. An attempt was made to determine the relationship between dimensional variation and nominal dimensions using a computer-aided design (CAD) file. A comparison between the dimensional deviations and the 3D fabricated pure PETG and all PETG/ATO composite specimens is presented via a statistical analysis in Figure 12a. The illustration of the PETG/6.0 wt.% ATO specimen, via color-coding, shows the results after comparing the structural deviations and the CAD data (Figure 12b,c).

In Figure 12d, the values of the A2N at a 95%-dimensional deviation of pure PETG and all the PETG/ATO samples are shown. The lowest value, below that of the pure PETG, was observed at PETG/6.0 wt.% (44.0% lower). Only PETG/10.0 wt.% ATO presents a higher value than that of pure PETG. The addition of the filler to the matrix material improved the dimensional deviation of the rest of the composites in relation to that of pure PETG, indicating a positive influence. At the same time, the composite with the highest dimensional accuracy also showed the highest overall mechanical performance among the materials tested, suggesting a possible connection between 3D printing quality and mechanical performance.

**Figure 13:**
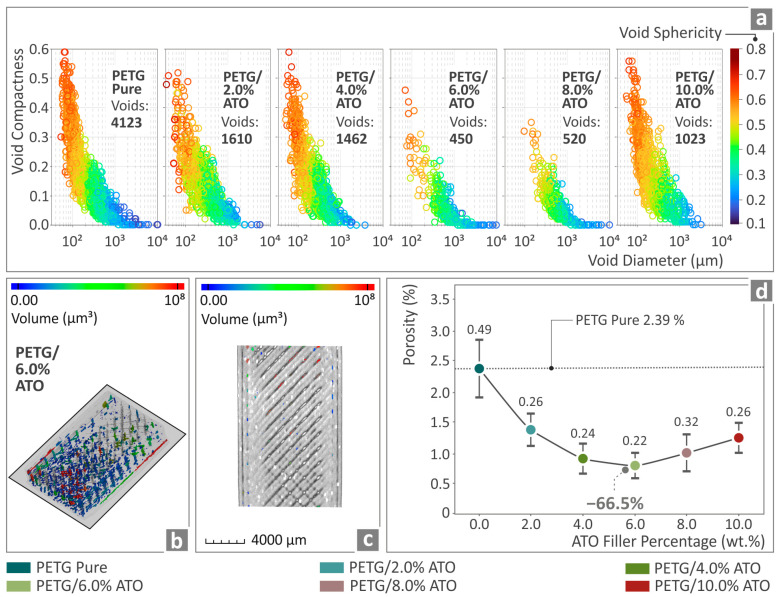
(**a**) Void graphs from the microcomputed tomography analysis of pure PETG and all the PETG/ATO composites, (**b**,**c**) visual representation of the PETG/6.0 wt.% ATO specimens’ porosity by color-coding mapping, and (**d**) porosity percentage of pure PETG and the PETG/x wt.% ATO, *x* = 2.0, 4.0, 6.0, 8.0, 10.0 wt.% composites.

The porosity results of the 3D fabricated specimens of pure PETG, PETG/2.0 wt.% ATO, PETG/4.0 wt.% ATO, PETG/6.0 wt.% ATO, PETG/8.0 wt.% ATO and PETG/10.0 wt.% ATO is presented in Figure 13. The void compactness and void sphericity of the void diameter graphs are shown in Figure 13a, whereas Figure 13b,c present the void distribution and respective volumes of the PETG/6.0 wt.% ATO. Figure 13d shows the values of the pure PETG and PETG/*x* wt.% ATO with *x* = 2.0, 4.0, 6.0, 8.0, 10.0 wt.% composites’ samples regarding the porosity percentage. It can be observed that the filler addition caused a decrease in the porosity of all the composites, especially in the case of PETG/6.0 wt.% ATO, where there was a 66.5% decrease in relation to pure PETG. Similar to the dimensional accuracy, the nanocomposite with a better internal 3D printing structure (6 wt.%) showed the highest mechanical performance.

### 3.8. SEM and EDS Mapping of the 3D-P Specimens

**Figure 14:**
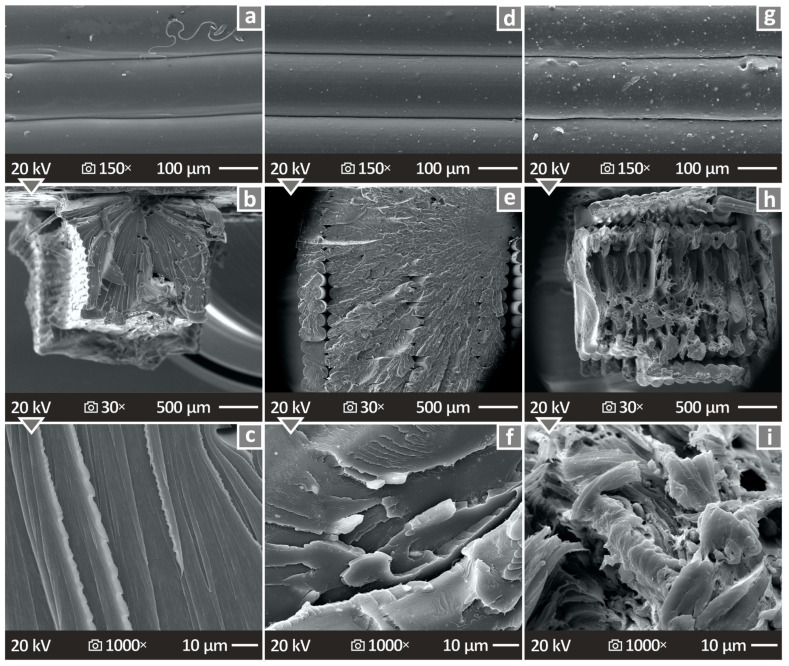
(**a**–**c**) Pure PETG SEM images of the side surface magnified at 150×, fracture surface magnified at 30× and 1000×, (**d**–**f**) PETG/4.0 wt.% ATO SEM images of the side surface magnified at 150×, fracture surface magnified at 30× and 1000×, and (**g**–**i**) PETG/8.0 wt.% ATO SEM images of the side surface magnified at 150×, fracture surface magnified at 30× and 1000×.

**Figure 15:**
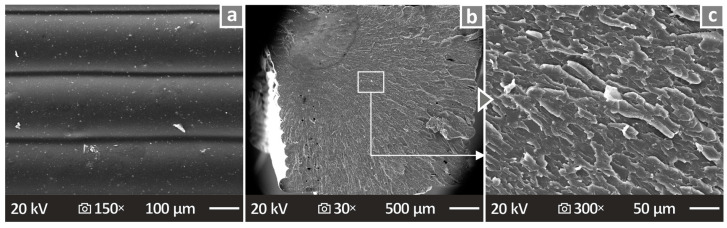
PETG/6.0 wt.% ATO SEM images (**a**) side surface magnified at 150×, (**b**) fracture surface magnified at 30×, and (**c**) fracture surface magnified at 150×.

Figure 14 and Figure 15 show the images obtained from SEM analysis of the sides and fracture surfaces of the fabricated 3D specimens. Figure 14a,d,g show the side surface images of the pure PETG/4.0 wt.% ATO and PETG/8.0 wt.% ATO, respectively, at 150× magnification. It can be seen that there are no defects, pores, or voids, and the fusion is well distributed. Figure 14b,e,h present the fracture surface images of the pure PETG/4.0 wt.% ATO and PETG/8.0 wt.% ATO, respectively, at 30× magnification. Figure 14c,f,i present the fracture surface images of pure PETG, PETG/4.0 wt.% ATO, and PETG/8.0 wt.% ATO, respectively, at 1000× magnification. The interlayer appeared to be smooth in pure PETG and PETG/4.0 wt.% ATO, while PETG/8.0 wt.% ATO presents a more brittle behavior. The pure PETG and PETG/4.0 wt.% ATO nanocomposite show a brittle fracture surface with minimum deviation. In most cases, a brittle behavior is presented in the SEM images, as no high deformation can be observed in the fracture surface at the low magnification levels or in the microscale. As the filler percentage in the composites increased to 8 wt.%, the fracture surface appeared to be more ductile with visible deformation at both magnification levels presented. Additionally, internal voids were visible, which can be attributed to the failure of the part. Figure 15 shows SEM images of the PETG/6.0 wt.% ATO, which showed the best mechanical response among the nanocomposites tested. As shown, the layer fusion and quality are excellent (Figure 15a). The fracture surface (Figure 15b,c) shows minimum deformation, indicating a brittle failure. The deformation is even less than the PETG/6.0 wt.% ATO, as shown in Figure 14. At the same time, a more solid 3D printing structure is presented in the PETG/6.0 wt.% ATO specimen, compared to the PETG/4.0 wt.% ATO one, with less internal voids. These features should have contributed to the superior mechanical performance of the specific nanocomposites (PETG/6.0 wt.% ATO) among the ones tested.

**Figure 16:**
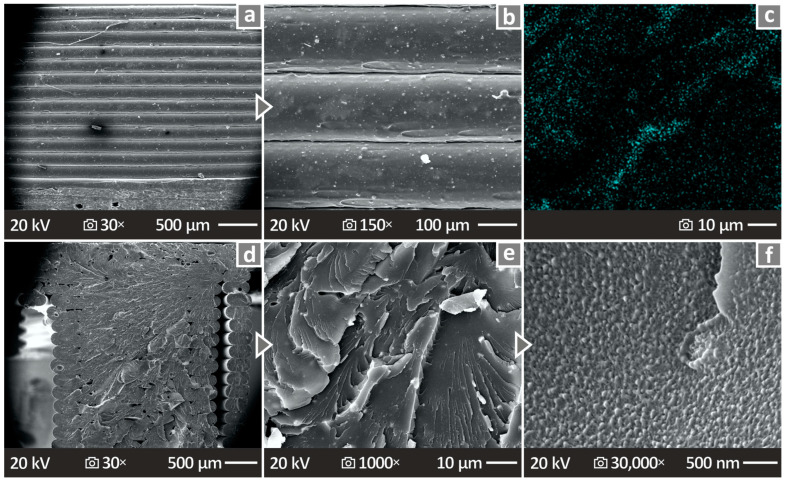
(**a**,**b**) Side surface images of PETG/10.0 wt.% ATO at 30× and 150× magnifications, respectively, (**c**) EDS mapping image from a chosen sample, (**d**–**f**) fracture surface images of PETG/10.0 wt.% ATO at 30× and 1000× and 30,000× magnifications, correspondingly.

Figure 16a,b present the SEM images of PETG/10.0 wt.% ATO side surface at 30× and 150× magnifications, respectively, where it can be observed that there are no voids and pores. Figure 16c shows an EDS mapping image of the antimony (Sb) element. The distribution of the elements in the observation area was not completely uniform, indicating possible particle clustering in this higher-filled nanocomposite. No particle clustering was observed with SEM or EDS at lower filler concentrations. Figure 16d–f show the SEM images captured from PETG/10.0 wt.% ATO fracture surface at 30×, 1000×, and 30,000× magnifications, correspondingly, where the revealed surfaces presented only a few defects.

## 4. Discussion

Five different PETG/*x* wt.% ATO were synthesized using five different filler percentages of *x* = 2.0, 4.0, 6.0, 8.0, and 10.0 wt.%. The mixtures were subsequently fed into an extruder to produce filaments that supplied the MEX 3D-P of the corresponding specimens. The thermal, rheological, mechanical, morphological, and electrical properties of the fabricated samples were studied. The mechanical performance of the specimens was investigated via tensile, flexural, Charpy impact strength, and M-H tests. SEM analysis was conducted on the side and fracture surfaces of the samples while they were dielectrically tested. Moreover, micro-computed tomography was conducted with the aim of comparing the initially designed models with the fabricated specimens.

**Figure 17:**
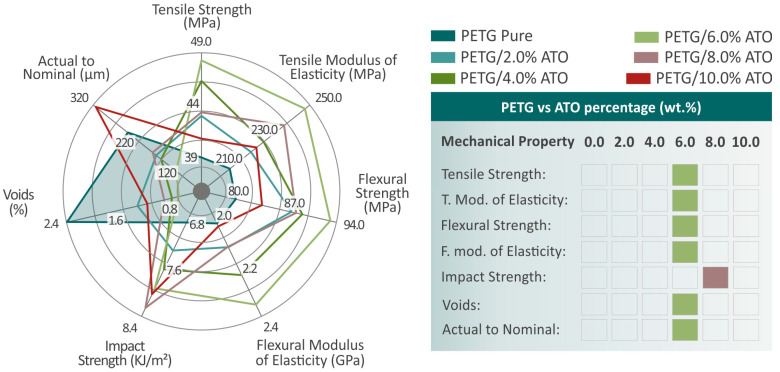
A spider-shaped graph of all the mechanical properties’ values is on the left area of the figure, and a board with all the mechanical properties matched with the composites that presented the most improved values is on the right area of the figure.

Overall, the mechanical properties of the specimens showed a significant improvement in relation to the performance of pure PETG with respect to all the different filler percentage composites, as shown in Figure 17 by observing the spider graph of the mechanical property values. In particular, for the case of PETG/6.0 wt.% ATO, almost all of the mechanical properties examined, except for the impact strength, were found to be at much higher levels than those of pure PETG, as also shown in the summarization board of Figure 17, where all the mechanical properties are matched with the composites that had the most improved values. The improvement in the mechanical properties of the nanocomposites up to 6.0 wt.% ATO content can be attributed to the mechanism behind enhancing the mechanical properties of polymeric matrices by incorporating nanoparticles. This mechanism revolves around the interactions between the nanoparticles and the matrix, as well as the restriction of polymer chain mobility due to the nanoparticles filling the voids between them [105,106,107,108,109,110,111,112,113]. At higher ATO loadings, the reduced mechanical properties of the nanocomposites can be attributed to the saturation of the ATO additive in the PETG matrix. The saturation of a filler in the matrix can have such an effect on the mechanical properties of the composites [108,114,115].

Regarding the electrical/dielectric behavior of the composites, no correlation is observed with respect to their mechanical performance with increasing the ATO filler content. In the range of ATO content used in the present study, no significant changes in either alternating current (AC)-electrical conductivity or dielectric permittivity were observed, and the observed variations are rather random, probably influenced by the randomly distributed ATO nanoparticles in the polymer matrix. A study on electrical properties of PMMA/ATO composites fabricated by compression molding at 170 °C [116] reported a considerable increase in electrical conductivity at very low ATO content, attributed to the segregated distribution of ATO nanoparticles along the edges of the transformed PMMA microspheres. This measured electrical percolation was achieved at 1% wt. ATO when monosize PMMA was used (90–106 μm) and at 1.5% wt. ATO, in the case of polydisperse PMMA with particle size in the range of 10–100 μm. In both cases, ATO particle size was 20–40 nm, similar to that used in the present case. However, in our case, the mechanical mixing of PETG pellets with ATO nanopowder and the subsequent extrusion procedure at 240 °C cannot result in such a uniform conductive network, and the percolation threshold may appear at higher filler concentrations. This discrepancy between mechanical performance and electrical enhancement of polymer nanocomposites has also been reported in the case of HDPE/Cu composites [117].

It should be noted that the SEM images presented very well-fabricated surfaces and well-distributed layering of the materials, even after the addition of the fillers. Very few voids were observed, and the defects were small. The addition of the ATO nanoparticles affected the rheological response of the PETG polymer. The overall viscosity decreased, and the MFR slightly increased. This showed no significant differences in the 3D printing quality, as the layer fusion, as presented in the images of the lateral surfaces, seemed to be rather intact even at higher-loaded nanocomposites. The layers also had a uniform thickness. By inspecting the 3D-printed internal structure on the μ-CT, the aforementioned assumption was verified. The addition of ATO improved the dimensional accuracy of the samples (only the highest-loaded nanocomposite showed inferior dimensional accuracy compared with the pure PETG sample) and reduced the number of voids. The nanocomposite that achieved the most improved mechanical performance had, at the same time, the lowest number of voids and better dimensional accuracy. This can safely lead to the assumption that better mechanical performance was achieved by the samples with better 3D printing quality. The addition of ATO made the samples more brittle because they failed at a lower strain than the pure PETG samples. However, when observing the fracture surfaces with SEM, it is shown that at the microscale, the higher loaded nanocomposites develop deformation before their failure, and the fracture surface seems to be collapsed when compared to the pure PETG and the nanocomposites with lower ATO content, in which a brittle fracture surface is presented in the SEM images.

An investigation of the thermal properties revealed that the addition of ATO negligibly affected the response of the PETG polymer at high temperatures. Simultaneously, it was verified that the temperatures used were safe for the nanocomposites and did not cause thermal degradation. SEM and EDS were also used to locate particle clustering in the fracture surfaces of the samples. Only the EDS of the higher-loaded composite revealed a non-uniform distribution of Sb, which is an indication of possible ATO nanoparticle clustering. This was not the case for nanocomposites with lower filler content, and it agrees with the reduced mechanical performance of the highest-loaded samples, which is attributed to the saturation of the ATO filler in the matrix. Even the highest-loaded samples showed slightly higher mechanical performance than pure PETG. The exact saturation threshold was not determined, as it was outside the scope of this research.

The distribution of the particles was examined in the high-magnification SEM images taken. The objective of these images was to detect any potential agglomerates within the materials and assess the dispersion quality of the nanofillers. Upon analyzing the fracture surface of the samples using SEM, it was observed that agglomerations were inevitable within the structure at the highest filler concentration in the nanocompound, as mentioned above. This observation was further supported by EDS analysis conducted in various regions of the fracture surfaces. Additionally, mechanical tests indicated that the deviation in results remained within acceptable limits, suggesting a consistent composition of the nanocompounds across all the samples examined. These findings and by considering the process followed for the mixing of the raw materials and the, specially designed for materials and additives mixing, extruder used, it can be safely assumed that a good distribution of the nanoparticles in the matrix was achieved.

The compatibility between the matrices and the additives in the literature can be estimated by correlating different properties derived during the characterization process [118,119]. Following the instructions of the literature, we can assume a good compatibility between PETG and ATO by considering the correlating different aspects of the study. The thermal properties of PETG were not significantly affected by the introduction of the ATO nanoparticles. Microscopy images on the outer surface of the filament revealed a smooth, defect-free surface; SEM images on the fracture surface of the samples did not present particle clustering for all of the filler concentrations tested except the highest one, in which SEM images and EDS mapping revealed particles aggregates in the samples. We have presented images up to 30,000×. Higher magnifications were not possible. The PETG was burned during the process at higher magnifications. At these magnifications, the nanoparticles are visible in the images, and they seem to be well dispersed and integrated with the matrix. Finally, if the additive is not compatible, it will make the composite weak [120], which is not the case here. The tensile strength was increased by 21%, and overall, the mechanical properties were improved. Additionally, the nanocomposites were prepared with a thermomechanical method, in which chemical interactions between the nanoparticles and the matrix are expected only in the contact area between them. Furthermore, it is not worth commenting on processability issues that were faced during the extrusion of the filaments and the 3D printing of the specimens.

Another aspect that is of great importance to the scientific community is the environmental implications of introducing nanocomposites in 3D printing. The release of nanoparticles during the preparation of the nanocomposites, during 3D printing, or the disposal of the samples is possible. This can lead to enhanced toxicity in the environment that can affect the ecosystem, especially in large amounts of nanoparticles since they then enter biological systems [121,122,123,124,125].

In a similar study [126], where polypropylene (PP) was combined with four concentrations of ATO (0.5–4.0 wt.%) and the fabricated samples underwent the same tests, a similar behavior was observed. Specifically, the addition of ATO as a filler improved the mechanical properties of pure PP, as also happened in the study herein. In the PP matrix, the addition of 0.5 wt.% ATO nanoparticles achieved the best mechanical performance in the tensile test, with an increase of about 7% compared to the unfilled PP. Herein, the maximum tensile strength value was achieved with higher ATO concentrations on the nanocomposites (6 wt.%), and the improvement was also significantly higher at 21%, compared to the unfilled PETG. In this [4] study, PETG was reinforced with carbon and aramid fibers to maximize its mechanical performance. The aim was to optimize the 3D printing parameters, and it is indicated that the 3D printing temperature is the most valuable parameter for optimizing the results. The 3D printing settings tested were similar to those of the current study in terms of layer height and nozzle temperature. The printing speed was higher in the current study than [4]. Comparing the results of the pure PETG between the two studies, the flexural strength found in the current study was about 25% higher than the [4] study, while the flexural modulus of elasticity was about 100% higher herein. On the other hand, the addition of CF in the [4] study improved the flexural strength by almost 100%, much higher than the 14.4% achieved by the addition of the ATO nanoparticles herein in the flexural strength. This higher improvement in the mechanical strength of the PETG polymer by the addition of carbon fibers is somehow expected, as carbon fibers usually impressively improve the performance of the matrices they are introduced. PETG has also been used with carbon to create composites, which are investigated in this [48] study for their mechanical properties. The results of the tensile test of the 3D-P PETG/CF samples showed a 23% improvement in yield strength. The 15.5% reported herein is a close value; still, the results it is not safe to be directly correlated as in this [48] study, the tests were carried out on prismatic specimens with different structural designs and not on standard specimens in accordance with the corresponding international standards. Overall, the results presented herein agree with the literature on PETG or ATO composites with similar reported response and reinforcement effects, while no research has presented the same composites to directly correlate the results.

## 5. Conclusions

In the present work, PETG was utilized and mixed with various weight percentages of ATO to create five different mixtures of composites, namely PETG/2.0 wt.% ATO, PETG/4.0 wt.% ATO, PETG/6.0 wt.% ATO, PETG/8.0 wt.% ATO and PETG/10.0 wt.% ATO, respectively. Subsequently, various filaments were produced and used to fabricate 3D-P specimens suitable for conducting mechanical tests. The tensile and flexural properties, Charpy impact strength, and microhardness of the samples were also examined. Overall, PETG/6.0 wt.% ATO showed the most improved mechanical performance of all different composites. Therefore, 6.0 wt.% ATO can be considered the optimum filler loading among the concentrations tested. This refers to the specific matrix material, that is, PETG, and for nanocomposites prepared using the approach followed herein and for 3D printed parts made with the MEX 3D printing method. Additionally, they underwent μ-CT and SEM analyses, and their broadband electric/dielectric behaviors were measured. The addition of ATO appears to have a negligible effect on the electrical properties of the nanocomposites, at least in the range of filler contents used in the present study. These results are related to those obtained from the respective tests conducted on pure PETG samples. Future studies could investigate PETG/ATO samples 3D printed under different settings, conditions, and 3D printing parameters, which could affect the final results, as shown in similar studies; in this study, the 3D printing parameters were optimized for the unfilled PETG polymer and used in the nanocomposites as well for comparison purposes. Therefore, the reinforcement achieved herein has margins for improvement. At the same time, the industrialization of the process can be investigated, considering additional aspects, such as the cost-efficiency of the process.

## Figures and Tables

**Table 1:** The significant Raman peaks, along with their related assignments regarding PP pure.

Wavenumber (cm^−1^)	Intensity	Raman Peak Assignment
631	Strong	phenyl ring vibration [88,89]
703	Medium	C–H out-of-plane bending [88]
772	Small	O–C(O)–O stretching [89]
793	Medium	
855	Strong	γ(C–OH)_ring_ [90,91]
1115	Strong	Skeletal vibrations, C–O–C bonds [92]
1172	Strong	Skeletal vibrations, C–O–C bonds [88,92]
1283	Strong	Skeletal vibrations, C–O–C bonds [88,92]
1369	Small	C–C–H, C–O–H, and O–C–H [92]
1409	Medium	C-H_3_ deformation [93]
1440–1464	Medium	C–H_3_ deformation [88,93]; C–H_2_ deformation [88,93]; C–H_3_ symmetric bending [89,93,94];
1613	Very Strong	Phenyl ring stretch [89]
1724	Very Strong	
2857	Medium	C–H_2_ symmetric stretching [92]
2890	Medium	CH_2_ symmetric stretching [92,95]
2955	Strong	CH_2_ asymmetric stretching [92]
3081	Strong	C-H stretching [93]

**Table 2:** Significant Raman peak differences in PETG/ATO samples from PETG/pure.

631	Gradual drop	Significant decrease
863	Gradual drop	Small decrease
1104	Gradual drop	Small decrease
1283	Gradual drop	Significant decrease
1613	Gradual drop	Significant decrease
1724	Gradual drop	Significant decrease
2955	Gradual drop	Significant decrease
3081	Gradual drop	Significant decrease

**Table 3:** Significant absorption peaks and their related assignments from PETG.

Wavenumber (cm^−1^)	Intensity	ATR/FTIR Peak Assignment
725	Strong	CH vibration [96]
792	Weak	Unidentified
872	Medium	CH vibration [96]
956	Medium	C–C stretching [97]
1016	Strong	CH_3_ rocking [98]; C–O–C asymmetric stretching [99]
1043	Weak	C–O stretching [97,99]
1093	Strong	C–O stretching [97]
1114	Strong	C–O–C–O–C symmetric vibration [98]
1173	Weak	CH_2_ and CH_3_ rocking [98]
1240	Strong	CH_3_ rocking [98]; C–O stretching [96,99]
1340	Weak	CH_2_ wagging vibration [97]
1371	Weak	CH_2_ wagging vibration [100]
1408	Medium	C–H bending [100]
1450	Medium	CH_2_ bending [97,100]
1504	Weak	CH vibration [96]
1578	Weak	C–N stretching [101]
1713	Strong	C=O vibration [96,98,99,100,102]
2850	Medium	CH symmetric stretching [97]
2923	Medium	CH asymmetric stretching [97]

**Table 4:** Significant ATR/FTIR peak differences in PETG/ATO samples from PETG/Pure.

Wavenumber (cm^−1^)	Change	Importance
668 (592–720)	Gradual increase	Significant
733	Gradual drop	Significant
1066	Gradual increase	Significant
1213 (1137–1301)	Gradual increase	Significant
1699 (1648–1738)	Gradual increase	Significant
2850	Gradual drop	Medium
2923	Gradual drop	Medium

## Data Availability

The raw/processed data required to reproduce these findings cannot be shared because of technical or time limitations.

## Supplementary Materials

The following supporting information can be downloaded at https://www.mdpi.com/article/10.3390/nano14090761/s1. Figure S1. (A–C) SEM images of ATO at 10,000×, 50,000×, and 100,000× magnifications, respectively, (D) EDS mapping image of ATO and (E) EDS analysis of ATO revealing its chemical composition.
