# Supplementary material for: Mechanical and Electrical Properties of Polyethylene Terephthalate Glycol/Antimony Tin Oxide Nanocomposites in Material Extrusion 3D Printing
Source: Nanomaterials (Basel). 2024 Apr 26;14(9):761. doi: 10.3390/nano14090761 (PMC11085549; doi:10.3390/nano14090761)
Supplement: Supplementary file 1 [file nanomaterials-14-00761-s001.zip › nanomaterials-2950890-supplementary.pdf]

nanomaterials

MDPI

Mechanical and Electrical Properties of Polyethylene Terephthalate Glycol/Antimony Tin Oxide Nanocomposites in Material Extrusion 3D Printing

Materials and methods

Figure S1A shows an SEM image of raw ATO at 10,000× magnification, and Figure A1B shows the indicated area of Figure S1A at 50,000× magnification. The highlighted area in Figure S1B was magnified 100,000× (Figure S1C). An EDS mapping image of ATO is presented in Figure S1D, and Figure S1E shows the results of EDS analysis of ATO, revealing its chemical composition. As expected, high levels of Sn were detected.

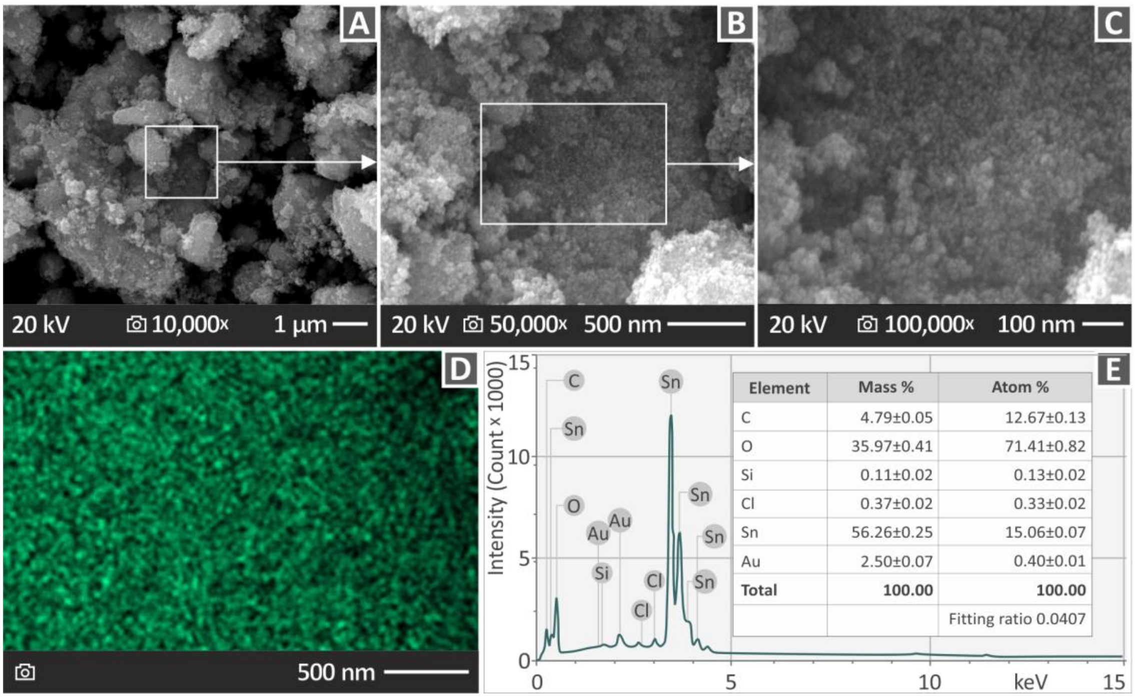

Figure S1. (A-C) SEM images of ATO at 10,000×, 50,000× and 100,000× magnifications respectively, **(D)** EDS mapping image of ATO and **(E)** EDS analysis of ATO revealing its chemical composition
